# Wellens Syndrome: prevalence, risk factors and coronary angiographic variation. A cross-sectional study

**DOI:** 10.1186/s12872-024-03752-y

**Published:** 2024-02-01

**Authors:** Sami Mohamed, Samoal Abdelaziz

**Affiliations:** 1 Department of Internal Medicine, Faculty of Medicine, Nile University, Khartoum, Sudan; 2 Department of cardiology, Ahmed Gasim Teaching Hospital, Khartoum North, Sudan

**Keywords:** Wellens Syndrome, Acute coronary Syndrome, Proximal LAD, Biphasic t wave, NSTEMI, Coronary angiography

## Abstract

**Background:**

Wellens syndrome complicates acute coronary syndrome and, if unmanaged, can lead to immanent myocardial infarction. This study aimed towards determining the prevalence of Wellens syndrome among acute coronary syndrome patients while focusing on both types and identifying the associated risk factors, then exploring the variation in affected coronary arteries within patients fulfilling Wellens syndrome criteria.

**Methods:**

Implementing a descriptive cross sectional hospital based observational study design, at Ahmed Gasim Teaching Hospital for Cardiac Surgery and Renal Transplantation in Khartoum North, Sudan, the study was conducted following using a non probability convenience sampling of patients fitting the inclusion criteria. Data was collected using closed ended structured questionnaires. Ethical clearance was obtained from relevant authorities. Statistical analysis was done using descriptive and comparative data analysis with the aid of the SPSS software, and STROBE guidelines were followed.

**Results:**

A total of 120 patients were included, 70 males and 50 females, majority in their fifth decade. 14 patients had no documented risk factors. 42.5% had STEMI, 34.2% had NSTEMI and 23.3% had unstable angina. Patients fulfilling Wellens syndrome criteria were 18 (15%), 55.6% of them were type A and 44.4% were type B. Most frequently encountered risk factor among Wellens syndrome patients was Diabetes (50%). Out of 16 Wellens syndrome patients who underwent coronary angiography, 50% had mid LAD involvement, most were type A; 25% had proximal LAD involvement and 25% had normal coronary angiography. There was some association between Wellens syndrome and NSTEMI, but no significant association with any specific risk factor.

**Conclusion:**

Wellens syndrome complicates 15% of acute coronary syndrome patients with a 55.6% possibility of becoming type A, it can present even without a specific predisposing risk factor and coronary angiographic variation other than the proximal part of the LAD artery may occur, including multiple vessels involvement.

**Condensed abstract:**

This is a descriptive cross sectional study conducted at Ahmed Gasim Teaching Hospital in Sudan, to determine the prevalence and risk factors of Wellens syndrome. Data was collected using questionnaires and analyzed with the SPSS software. Out of 120 patients, 14 patients had no documented risk factors. 34.2% had NSTEMI and 23.3% had unstable angina. Patients fulfilling Wellens syndrome criteria were 18 (15%). The commonest risk factor among Wellens syndrome patients was Diabetes (50%). 50% of Wellens syndrome patients had mid LAD involvement. The study concluded that Wellens syndrome is not rare, it can present without specific risk factor and coronary angiographic variation other than the proximal LAD artery can occur.

**Supplementary Information:**

The online version contains supplementary material available at 10.1186/s12872-024-03752-y.

## Introduction

Wellens syndrome was first described in 1982 by Wellens, H J and others [1], as a characteristic electrocardiographic pattern indicating a critical stenosis high in the left anterior descending coronary artery (LAD), which is at high risk for the development of an extensive anterior wall myocardial infarction if left untreated. This specific pattern was later found to be prevalent in about 14 to 18% of patients presenting with acute coronary syndrome and represents both a sensitive and a highly specific indicator for significant proximal left anterior descending artery (LAD) stenosis [2]. Although several cases were found to have other coronary arteries alongside or other than the LAD [3], with variation on the associated number of vessels affected [4], while others showed mildly affected LAD suggesting arterial spasm [5]. Those cases varied in different studies.

**Fig. 1:**
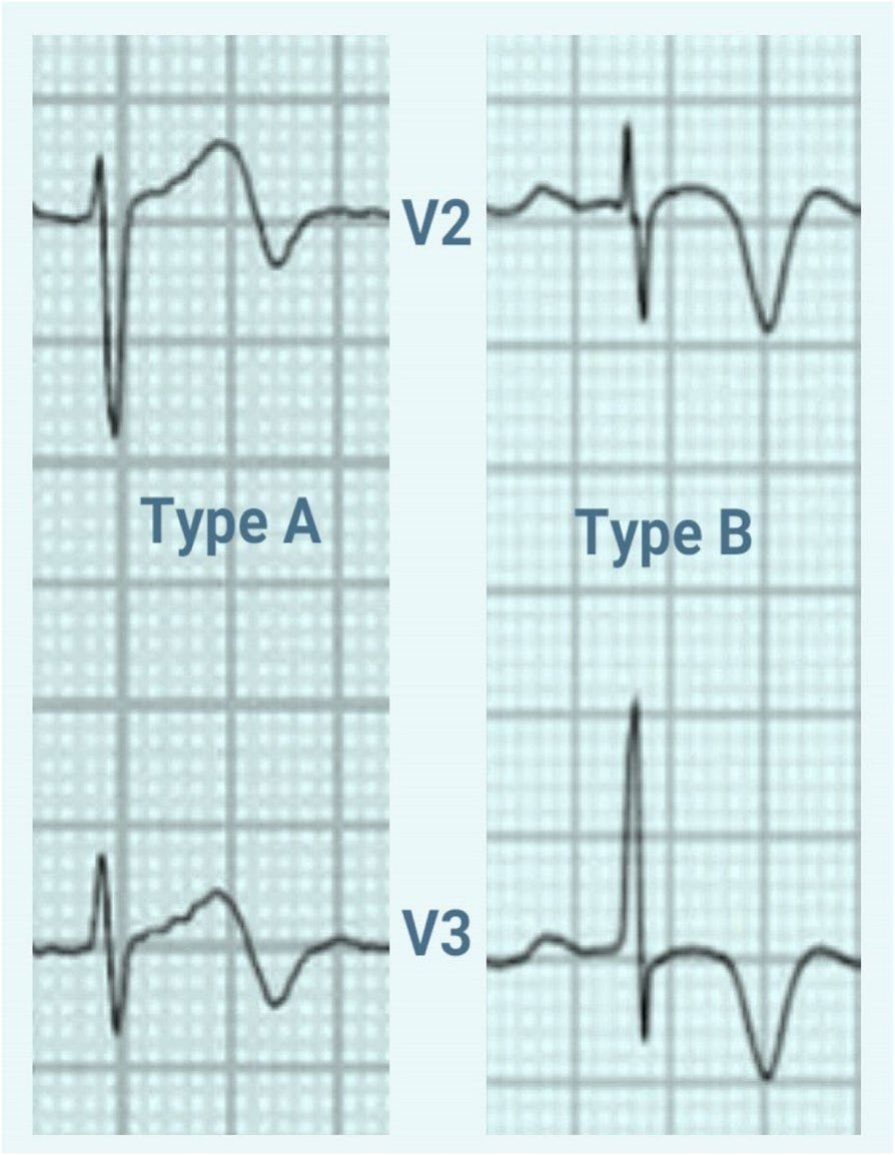
ECG patterns showing criteria and types of Wellens syndrome

There are two types of Wellens syndrome based on the electrocardiographic (ECG) finding (Fig. 1), though some have reversed this description [1, 6]:

- Type A biphasic T-waves in the pericordial leads V2 and V3.
- Type B Deeply inverted T-waves in the pericordial leads V2 and V3

The following are the required criteria for the diagnose of Wellens syndrome [6, 7]:

- Prior history of chest pain.
- During chest pain: ECG is normal or with mild ST elevation or depression, or with terminal negative deflection of the T wave in V_1_ and V_2_.
- Cardiac enzymes are normal or mildly elevated. No pathologic precordial Q-waves.
- No loss of precordial R-waves.
- Deeply inverted or biphasic T-waves in V2 and V3, possibly V1, V4, V5 and/or V6 when pain free [8].

The challenge in diagnosing this syndrome is that it occurs namely during chest pain free periods. However, some cases were encountered without history of chest pain [9], though a case was reported presenting within the initial chest pain period [10].

Apart from aforementioned criteria, other prognostic predictors related to acute anterior myocardial infarction, a usual sequela to LAD involvement, were later investigated. A pilot study suggested that calculation of electrocardiographic total Q wave amplitude/precordial total R wave amplitude ratio in patients with established acute anterior myocardial infarction, potentially serves as an independent prognostic factor for in-hospital mortality [11]. Higher ratios were also linked to no-reflow outcomes in those patients [12]. Similarly, development of ST segment elevation in inferior leads, rather than ST segment depression, in patients previously diagnosed with acute anterior myocardial infarction, was previously found to be associated with higher mortality as well [13]. This sheds light on the paramount importance of prompt recognition of Wellens syndrome as a preventive measure.

Since Wellens syndrome is considered a subcategory of acute coronary syndrome, risk factors for its development are equivalent to those of coronary artery disease [14]. They include hypertension, diabetes, hyperlipidemia, previous coronary artery events, cigarette smoking, hormone replacement therapy, chronic kidney disease [15, 16], family history and even genetic predisposition [17]. These risk factors vary in different populations with cigarette smoking being in the lead.

This study focuses on the prevalence of Wellens syndrome among acute coronary syndrome patients, while trying to identify the most prevalently associated predisposing risk factors.

Wellens syndrome is a serious condition that in most cases needs revascularization [8], and can progress rapidly in a matter of a few days, or less, to imminent myocardial infarction [18]. Delayed intervention during this narrow window can have fatal, yet predictable, consequences [19]. Unfortunately it is often overlooked [20]. Therefore, it is essential to study this syndrome in order to ensure effective prevention and proper management.

Most of health care professionals, including junior doctors, can usually identify patients with acute coronary syndrome. Yet Wellens syndrome is under recognized. Therefore, studying its prevalence can help draw more attention to this serious condition. As well as raising awareness to encourage early identification.

Furthermore, few studies stratified the association of risk factors to the syndrome [21]. And knowledge of risk factors variation in different populations is essential for implementation of preventive measures.

## Objectives

1. To determine the prevalence of Wellens syndrome in patients with acute coronary syndrome.
2. To identify the most common type of Wellens syndrome.
3. To determine the most associated risk factor with Wellens syndrome.
4. To identify the commonest coronary artery affected in patients with Wellens syndrome.

### Overview of literature

Wellens syndrome was first described in 1982 [1]. It revealed characteristic electrocardiographic pattern indicating a critical stenosis high in left anterior descending coronary artery (LAD) in patients admitted because of impending myocardial infarction. These patients, who showed characteristic ST-T segment changes in the precordial leads on or shortly after admission, had critical stenosis high in the LAD. Of the patients consecutively admitted because of unstable angina, 18% showing this ECG pattern, suggesting that this finding is not rare. Majority of patients who did not undergo coronary angiography eventually had massive anterior myocardial infarction. Therefore, those who present with these ECG findings require prompt coronary angiography with possible need for intervention.

The development of new T-wave inversion in patients with unstable angina was reported in 1983 [18]. The electrocardiograms of the patients were correlated with coronary angiographic findings. Most patients had greater than or equal to 70% diameter reduction of the LAD. The sensitivity of T-wave inversion for significant LAD stenosis was 69%, specificity 89%. Therefore, development of new T- wave inversion greater than or equal to 2 mm in patients with unstable angina is predictive of significant coronary artery stenosis, and constitues poor prognosis if treated medically.

A larger scale study in 1989 [2] included 1260 unstable angina patients, and found that 14% had the characteristic Wellens ECG pattern. Most had these findings at presentation, while the rest developed them after admission. Coronary angiographic findings revealed atleast 50% proximal LAD stenosis in all patients. Few patients had total LAD occlusion in addition to collaterals.

An article previously described Wellens syndrome as a pattern of electrocardiographic T-wave changes associated with critical proximal LAD [7]. The syndrome was also referred to as LAD coronary T-wave syndrome. It similarily described the syndrome’s criteria. The persistence of T-wave abnormalities that may last for several days was also reported.

An interesting study was published in Pakistan [4] describing the coronary angiographic findings in patients with unstable angina showing biphasic inversion of T-waves in precordial leads on electrocardiogram, in 100 patients showing the characteristic electrocardiogram pattern. Its Angiographic findings revealed that 50 (50%) patients had coronary artery stenosis in the proximal part of the left anterior descending artery, while 22 (22%) showed the occlusion in the middle segment. And two vessel disease was most commonly observed during cardiac catheterisation.

Another smaller study was published in India [21], taking into consideration risk factors variation, included a total of 40 patients. Patients suffered from hypertension, diabetes mellitus, dyslipidemia, smoking and history of premature coronary artery disease. Majority had single vessel disease (SVD) with LAD involvement, while some had double vessel disease (DVD) and minority had normal coronary arteries.

In view of prevalence, an article aimed to study its prevalence, clinical significance and prognostic value in patients with non-ST elevation myocardial infarction (NSTEMI) [3]. Analysis of 481 consecutive patients presenting with NSTEMI who underwent coronary angiography categorized patients into a Wellens group and control group. 4.2% had Wellens syndrome. Patients had varying coronary artery involvement, but most had LAD pathology, with no significant prognostic predictions after group comparison.

Several case reports were published over the past few years describing typical and atypical presentations. With different types of the syndrome [22]. Some described typical cases of young male patients [6, 23].

Other reports described atypical cases such as patient presenting with syncope without chest pain [9]. And a reported case of Wellens syndrome in a patient with cocaine abuse [24].

A case of a patient’s coronary angiography revealed moderate stenosis in proximal LAD, and coronary artery spasm was suggested [5]. Also in another case unlike the classic Wellens syndrome, which needs aggressive coronary intervention, the patient showed favorable prognosis with conservative medical therapy [25].

A reported case of a patient with previous chest pain, whose ECG showing subtle ischemic changes, was initially overlooked [26]. But a repeat ECG taken during the painless period showed a biphasic T wave, suggestive of Wellens syndrome, which was followed by an immediate coronary angiogram.

Other cases reported highlighted associated risk factors such as smoking, diabetes or previous history of coronary artery disease, and named the syndrome 'the widow maker' [20, 27].

The youngest reported case of Wellens syndrome [28] was a 24 years old female, who was initially unrecognized and progressed to non ST elevation myocardial infarction (NSTEMI). The patient was found to have critical narrowing of the proximal LAD which was successfully treated with a drug eluting stent. The syndrome was reported occurring in an elderly patient as well [26].

Wellens syndrome has also been identified in a patient with a small plaque in the proximal LAD, with slow flow in the LAD detected by coronary angiography suggesting arterial spasm [5].

In regards to risk factors to developing acute coronary syndrome, which includes Wellens syndrome, in 1984 the Multiple Risk Factor Intervention Trial screening program [29] studied the association of diastolic blood pressure level, serum cholesterol concentration, and cigarettes per day with mortality. The relationship of serum cholesterol concentration and reported cigarettes per day to CHD, and cerebrovascular disease mortality was similar for black and white males, and the lower coronary heart disease mortality among black males compared to white males was most apparent among hypertensive males.

Later, a multinational study took place in 2005 [14] involving 103 hospitals in 25 countries in Europe and the Mediterranean basin. The study included 10,253 patients with acute coronary syndrome (ACS). Smokers had an increased risk to present with STEMI. Hypertension, diabetes and high body mass index (BMI) were less associated.

Recently, a larger scale study was conducted in 2022 [29, 30] highlighting a Wellens syndrome incidence of 5.7% among 3528 ACS patients. NSTEMI was more frequently encountered, and early coronary angiography was more prominent among Wellens syndrome patients. The study found that Wellens syndrome was not associated with poor prognosis, except in the presence of specific risk factors such as diabetes, advance age and chronic kidney disease.

## Materials and methods

### Study design

This was a descriptive cross-sectional hospital based observational study. Guidelines from the STROBE Statement for observational, and for cross-sectional studies was followed during implentation.

### Study area

Ahmed Gasim Teaching Hospital for Cardiac Surgery and Renal Transplantation, Khartoum North, Sudan.

### Study population

The study population included all patients in view of the case definition, inclusion and exclusion criteria.

### Case definition

Any adult patient of any gender who presented to the study area and is diagnosed with acute coronary syndrome of any type.

### Inclusion criteria

Any patient according to the case definition.

### Exclusion criteria

- Patients where data about their risk factors were unable to be obtained or is incomplete.
- Patients who did not have an electrocardiogram (ECG) recorded during a pain free period.
- Patients who was unable to provide an informed consent.

### Sampling

The study was conducted using a non probability convenience sampling, using total coverage of any patient who fit the inclusion criteria inside the study area, during the period between April 2017 and September 2017. sample size was 120, and was calculated using standard statistical formula with a 95% confidence level, a 50% population proportion, a 5% margin of error, and a population size of 174.

### Data collection methods and tools

Data was collected using closed ended structured questionnaires, attached with documented guidance on identification of Wellens syndrome with example ECG tracings. They were filled by previously taught and trained data collectors, who are medical doctors capable of identifying the patients according the case definition, as well as Wellens syndrome. Wellens syndrome criteria was obtained from the patient’s electrocardiogram (ECG) done in chest pain free periods. All ECGs done for patients with acute coronary syndrome were observed during the period of hospital stay. Clinical data regarding risk factors was taken from the patient through short interviews and with the use of confirmed clinical records or laboratory investigations as appropriate. Coronary angiographic data, if present, was obtained from direct observation of the procedure with the aid of the operating cardiologist, or through a coronary angiography report performed and signed by a certified cardiologist.

### Data management

Data were revised, coded and sorted into a master sheet. According the study variables and with the aid of an approved statistician, descriptive data analysis was done using the mean, median, standard deviations, tables, charts and frequency distribution. As well as comparative statistics using cross tabulations, Chi square testing and *p* value calculation, with a confidence interval of 95%. The SPSS software was used as a data management and analysis tool.

## Results

### Patients’ pathway, data inclusion and selective analysis

**Fig. 2:**
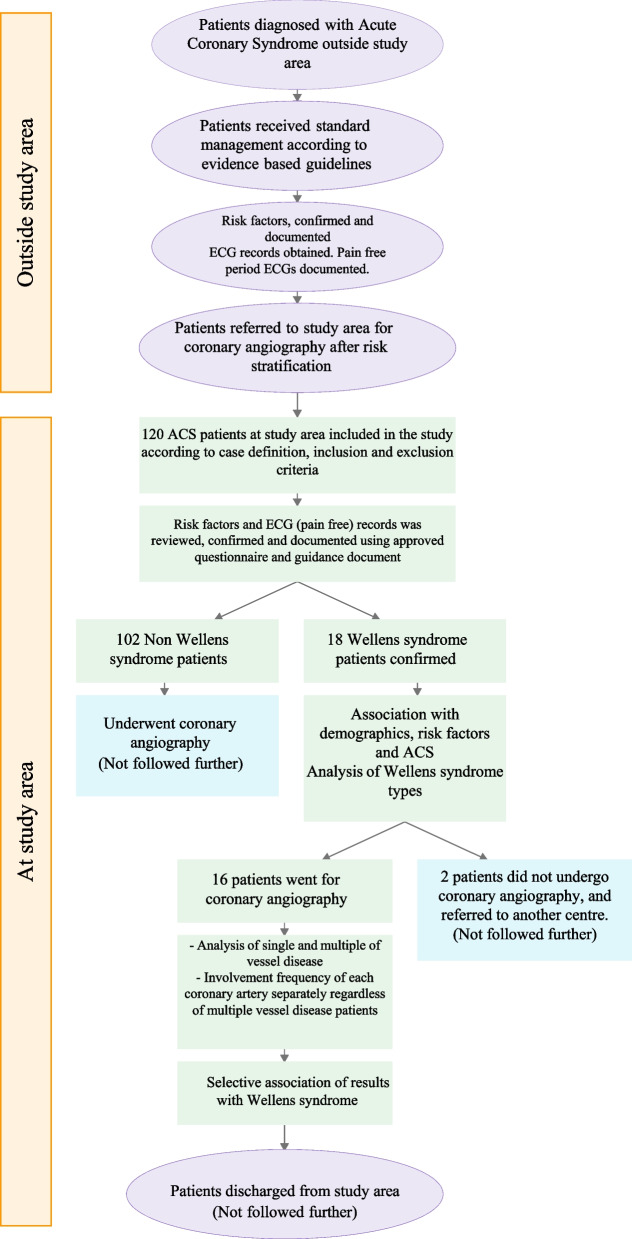
Flow diagram of patients’ pathway throughout the course of the study

A flow diagram for patients’ pathway is illustrated in (Fig. 2). All patients included in the study were diagnosed with ACS on presentation at different emergency departments outside the study area by following either NICE, American Heart Association, European Resuscitation Council, European Society of Cardiology or national guidelines. They received standard treatment for ACS in accordance with latest evidence based recommendations from aforementioned guidance, including optimal medical treatment. They were later referred to the study area, being the nearest cardiac centre with interventional facilities, for coronary angiography, either within hours from emergency departments, or within days from respective coronary care units depending on their risk stratification according to standard scoring systems, namely GRACE or equivalent. All confirmatory supporting documentation were reviewed by data collectors, described earlier, before inclusion in the study.

Results were obtained through the final analysis of 120 patients who fit the inclusion criteria of the study, taking into account the demographical distribution of data as well as the objectives of the study. There were no missing data in regards to study variables. ECG tracing of all patitents were reviewed and confirmed to have been recorded during pain free periods before approval. This included all those who fulfilled Wellens syndrome criteria.

Coronary angiographic findings were followed in all patients who fulfilled Wellens syndrome criteria. It is Relevant to mention that two out of the total 18 Wellens syndrome patients did not undergo coronary angiography. One of the two had difficulty in arterial line cannulation and was referred to another center outside the study area. The other patient was young and had no identifiable risk factor for coronary artery disease, and was recommended for none invasive observational approach after risk stratification and was refered to another center as well. Both were excluded from data analysis in regards to angiographic findings among Wellens sysndrome patients. Thus, this specific part of statistical analysis was performed on the 16 patients who underwent coronary angiography. Furthermore, descriptive analysis of affected coronary arteries included every single artery within each multiple vessel disease Wellens syndrome patients as an isolated variable. This was carried out in order to associate each coronary artery with Wellens syndrome regardless of multiple vessel disease status which may be caused by either Wellens syndrome or other concomitant pathological process.

### Demographics

Out of the 120 patients, 70 (57.3%) were males and 50 (41.7%) were females. Most patients were in their fifth decade (25.8% in the range of 50–59 years), followed closely by the seventh decade (25% in the range of 70–79 years) (Table 1).

**Table 1:** Frequency of demographical data, risk factors for developing ACS, ACS type prentation, patients who fulfilled Wellens syndrome criteria and types of Wellens syndrome

Parameter	Data	Frequency (out of 120)	Percentage (out of total)
Gender	Male	70	58.30%
	Female	50	41.70%
Age	20–29 years	3	2.50%
	30–39 years	5	4.20%
	40–49 years	22	18.30%
	50–59 years	31	25.80%
	60–69 years	29	24.20%
	70–79 years	30	25%
Risk factors	Hypertension	55	46%
	Diabetes	54	45%
	Hyperlipidemia	17	14.20%
	Family history of coronary artery disease	37	30.80%
	Previous coronary artery disease	16	13.30%
	Chronic kidney disease	1	0.80%
	Smoking	27	22.50%
Acute coronary syndrome presentation	STEMI	51	42.50%
	NSTEMI	41	34.20%
	Unstable angina	28	23.30%
Wellens syndrome criteria fulfilled	Yes	18	15%
	No	102	85%
Type of Wellens syndrome	Type A (biphasic T wave in V2-V3)	10	6.70% (55.6% of Wellens)
	Type B (inverted T wave in V2-V3)	8	8.30% (44.4% of Wellens)

### Risk factors

Majority of patients (106, 88.3%) had risk factors for the development of acute coronary syndrome, and only 14 patients (11.7%) had no documented risk factors with the exclusion of age and gender variation (Table 1). The commonest risk factor was hypertension (45.8%), followed closely by diabetes (45.0%). The least prevalent risk factor was chronic kidney disease (one patient, 0.8%). Relevant to mention that total number of cigarette smoking patients were 27 (22.5%), most of which (92.6%) were males.

### Types of acute coronary syndrome

All 120 patients fulfilled the inclusion criteria and were diagnosed with acute coronary syndrome. 51 patients (42.5%) were diagnosed with ST elevation myocardial infarction (STEMI), while 41 (34.2%) where cases of none ST elevation myocardial infarction (NSTEMI), and the rest (28, 23%) were unstable angina patients. (Table 1).

### Prevalence of Wellens syndrome

The total number of patients fulfilling criteria of Wellens syndrome was 18 patients (15%) (Table 1), 10 of which (55.6%) were type A (biphasic T waves in V2-V3), and 8 (44.4%) were type B (T wave inversion in V2-V3) (Table 1).

Out of the 18 Wellens syndrome patients, 12 (66.7%) were diagnosed as NSTEMI, the rest (6, 33.3%) were unstable angina patients (Table 1). None of the STEMI patients fulfilled Wellens syndrome criteria.

The 12 NSTEMI patients who fulfilled Wellens syndrome criteria represented 29.3% out of total NSTEMI patient, while the 8 unstable angina patients represented 21.4% out of unstable angina patients (Pearson Chi-Square 16.454, *P*-value < 0.01) (Table 2).

**Table 2:** Association of Wellens syndrome with demographical variation, risk factors, ACS type and coronary angiographic findings

Parameters		Patients fulfilling Wellens syndrome criteria (18-patients)		Statistical significance	
		Frequency	Percent (out of 18 patients)	Pearson ChiSquare	*p*-value
Gender	Male	11	61.10%	0.21	0.79
	Female	7	38.90%		
Age	20–29 years	1	5.56%	3.06	0.69
	30–39 years	0	0%		
	40–49 years	3	16.70%		
	50–59 years	5	27.80%		
	60–69 years	6	33.33%		
	70–79 years	3	16.70%		
Acute coronary syndrome type	STEMI	0	0%	16.45	0
	NSTEMI	12	66.70%		
	Unstable angina	6	33.30%		
Risk factors	Hypertension	7	38.90%	–	–
	Diabetes	9	50%	0.21	0.64
	Hyperlipidemia	3	16.70%	–	–
	Family history of coronary artery disease	5	27.80%		
	Previous coronary artery disease	1	5.56%		
	Chronic kidney disease	1	5.56%		
	Smoking	3	16.70%		
	No risk factor	2	11.10%		
Coronary angiography: Involvement frequency of each coronary artery (displayed as isolated discrete nominal variables)	Normal coronary arteries	4	25% (out of 16 patients)	–	–
	Left main coronary artery	1	6.30% (out of 16 patients)		
	Proximal left anterior descending artery	4	25% (out of 16 patients)		
	Mid left anterior descending artery	8	50% (out of 16 patients)		
	Diagonal artery	1	6.30% (out of 16 patients)		
	Left circumflex artery (at any site)	3	18.80% (out of 16 patients)		
	Right coronary artery (at any site)	4	25% (out of 16 patients)		
	Not done	2	–		
Coronary angiography: Number of vessels affected	Single vessel disease	10	62.50% (out of 16 patients)		
	Two vessel disease	4	25% (out of 16 patients		
	Three vessel disease	2	12.5% (out of 16 patients)		

### Type of Wellens syndrome

Among patients who were type A Wellens syndrome (10 patients), 6 were NSTEMI patients (60%) and 4 were unstable angina patients (40%). On the other hand, among the 8 type B patients, 6 were NSTEMI patients (75%) and 2 were unstable angina patients (25%) (Pearson Chi-Square 0.45, *P*-value 0.5) (Table 3).

**Table 3:** Association of Wellens syndrome types with ACS types and coronary angiographic findings

Parameters		Type of Wellens syndrome				Statistical significance	
		Type A (biphasic T wave in V2-V3)		Type B (inverted T wave in V2-V3)			
		Frequency (out of 18 patients)	Percent (out of 18 patients)	Frequency (out of 18 patients)	Percent (out of 18 patients)	Pearson ChiSquare	*p* value
Acute coronary syndrome presentation	STEMI	0	0%	0	0%	0.45	0.5
	NSTEMI	6	33.33%	6	33.33%		
	Unstable angina	4	22.22%	2	11.11%		
Coronary angiography outcome	Normal coronary arteries	2	12.50% (out of 16 patients)	2	12.50% (out of 16 patients)	–	–
	Left main coronary artery	1	6.25% (out of 16 patients)	0	0% (out of 16 patients)		
	Proximal left anterior descending artery	2	12.50% (out of 16 patients)	2	12.50% (out of 16 patients)		
	Mid left anterior descending artery	6	37.50% (out of 16 patients)	2	12.50% (out of 16 patients)		
	Diagonal artery	1	6.25% (out of 16 patients)	0	0%		
	Left circumflex artery (at any site)	2	12.50% (out of 16 patients)	1	6.25% (out of 16 patients)		
	Right coronary artery (at any site)	3	18.75% (out of 16 patients)	1	6.25% (out of 16 patients)		
	Not done	0	0%	2	12.50% (out of 16 patients)		

### Risk factors of Wellens syndrome

Out of the 18 Wellens syndrome patients, there were 2 patients who had no identifiable risk factor for the development of acute coronary syndrome. Nine patients (50%) were diabetics, 7 (38.9%) had hypertension, 5 (27.8%) had family history of coronary artery disease, 3 (16.7%) had Hyperlipidemia, 3 (16.7%) were smokers and only 1 (5.56%) had previous history of coronary artery disease as well as one patient (5.56%) had chronic kidney disease (Table 2).

Six Wellens syndrome patients were in their sixth decade, representing the highest frequency (33.3%) of total Wellens syndrome patients, and one patient was in the second decade representing the lowest. None of Wellens syndrome patients were in their third decade. (Pearson Chi-Square 3.06, *P*-value 0.69) (Table 2).

Most Wellens syndrome patients were males, representing 61.1% (11 patients). Female patients represented 38.9% (7 patients). (Pearson Chi-Square 0.067, P-value 0.795) (Table 2).

The most frequent risk factor was diabetes, representing 50% of Wellens syndrome patients. (Pearson Chi-Square 0.21, P-value 0.64) (Table 2). The least frequent risk factors were previous coronary artery disease and chronic kidney disease; each was present in only one patient.

### Affected coronary arteries

Among the 16 Wellens syndrome patients who underwent coronary angiography, 10 (62.5%) had single vessel disease, 4 (25%) had two vessel disease and 2 (12.5%) had three vessel disease (Table 2). Half of the sixteen patients (8, 50%) had an affected Mid left anterior descending artery, 4 (25%) had proximal LAD artery involvement, 4 (25%) had none isolated right coronary artery involvement, 4 (25%) had normal coronary angiography, 3 (18%) had left circumflex artery involvement, one patient (6.3%) had an affected left main coronary artery and one patient (6.3%) had diagonal artery involvement (Table 2). None of Wellens syndrome patients had distal left anterior descending artery involvement.

Out of the 10 type A Wellens syndrome patients who underwent coronary angiography, 6 (37.5%) had mid LAD involvement, and 2 patients (12.5%) had proximal LAD artery involvement. While among type B Wellens syndrome patients, both proximal and mid LAD artery were affected in 2 (12.5%) patients respectively (Table 3).

## Discussion

This descriptive cross sectional study provided supportive evidence to more enlightenment about Wellens syndrome with special regards to its prevalence, risk factors and potential variation in coronary angiographic findings.

Studying of Wellens syndrome risk factors and angiographic findings was reinforced by the acceptable prevalence which was encountered, though being an uncommon condition may affect the ability of results to represent different populations in general.

### Demographics

Out of the total population fulfilling the inclusion criteria (120 patients), there was no significant variation in regards to gender, though males were slightly more frequent than females (Table 1).

Descriptive analysis revealed two age peaks in prevalence of acute coronary syndrome, namely fifth and seventh decades. While seventh decade peak is expected, the fifth decade peak suggests presence of more risk factors that would explain its frequency.

### Risk factors

Out of total acute coronary patients, only 14 patients had no identifiable risk factor after excluding age and gender. Acute coronary syndrome in many cases was reported in patients with no known risk factors.

The commonest two risk factors encountered were Hypertension (45.8%) and Diabetes (45%), with Hypertension being slightly more frequent. Smoking represented only (22.5%). And there was only one patient who had chronic kidney disease. These findings slightly deviate from the multinational study [14] that highlighted smoking as the most associated risk for acute coronary syndrome especially STEMI. It is acceptable to suggest that prevalence of both diabetes and hypertension is higher than smokers who are complicated by acute coronary syndrome in the general population where the study was conducted. Moreover, gender distribution among smokers revealed significant variation suggesting that males are more associated with cigarette smoking than females (*p*-value < 0.01). This would contribute to the lower incidence of acute coronary syndrome specifically due to smoking among females.

### Types of acute coronary syndrome

Most patients were diagnosed with STEMI (42.5%), while NTSEMI and unstable angina represent 34.2 and 23.3% respectively (Table 1).

### Prevalence of Wellens syndrome

The study revealed that the overall prevalence of Wellens syndrome among acute coronary syndrome is 15% regardless of acute coronary syndrome type (Table 1). Thus answering one of the main study questions. This finding is similar to previous studies that found a prevalence of 14 and 18% in different populations [1, 2]. However, those studies had an inclusion criteria of unstable angina only. Furthermore, a study published later [3] found a prevalence of 4.2% but among NSTEMI patient alone.

Further analysis of prevalence with cross tabulation for each type of acute coronary syndrome revealed that NSTEMI Wellens syndrome patients constituted 29.3% of the total NSTEMI patients. While unstable angina Wellens syndrome patients constituted 21.4% of total unstable angina patients. (Pearson Chi-Square 16.454, *P*-value < 0.01). Which suggests that Wellens syndrome mat be more associated with NSTEMI, and its frequency is not rare, though statistical analysis of such small subgroups may not represent larger populations.

None of the STEMI patients fulfilled Wellens syndrome criteria. This supports previous evidence that Wellens syndrome occurs mainly in NSTEMI and unstable angina. Interestingly enough, 4 STEMI patient partially fulfilled Wellens syndrome criteria with the exception of presence of Q waves. And therefore, they were excluded from further analysis and they were not considered as Wellens syndrome patients. However, their coronary angiographic findings were followed only out of interest. Their results revealed proximal LAD involvement in all four patients. A recently published case reported type A Wellens syndrome with conconmitant COVID-19 pneumonia. The highlighted difficulty in management was complicated by STEMI, and a subsequent coronary angiography revealing the classical proximal LAD artery involvement [31]. Moreover, recently reported cases also highted COVID-19 association as well [32]. This may lead to rethinking the possibility of Wellens syndrome as the precurser of the four aforementioned STEMI patients.

### Type of Wellens syndrome

Analysis of the 18 Wellens syndrome patients revealed that 55.6% were type A and 44.4% were type B, the later being more frequent (Table 1). Furthermore, cross tabulation of Wellens syndrome types with acute coronary syndrome types showed that NSTEMI is the most associated acute coronary syndrome with both type A and type B equally, representing 66.6% out of all Wellens syndrome patients. However, statistical analysis suggests that this association is not strongly significant (*p*-value > 0.05) (Table 3).

### Risk factors of Wellens syndrome

Despite having no identifiable risk factors, 2 out of the 18 Wellens syndrome patients developed acute coronary syndrome and fulfilled Wellens syndrome criteria (Table 2), suggesting that Wellens syndrome can occur, though uncommon, without apparent risk factors resembling acute coronary syndrome in that manner. Moreover, the most frequently encountered risk factor among Wellens syndrome patients was Diabetes (50%) followed by Hypertension (38.9%), with the former being more frequent (Table 2). This supports a few case report with Wellens syndrome patient being patients with diabetes [20, 27]. But deviates from another study that highlighted Hyperlipidemia as a more frequently encountered risk factor in Wellens syndrome [29]. This may be due to variation of acute coronary syndrome risk factor profiles in different populations. However, analysis suggests that the association is not strongly significant (*p*-value> 0.05). Moreover, there were no statistically significant association between Wellens syndrome and age nor gender, despite sixth decade (33.3%) and male gender (61.1%) being most frequently encountered (Table 2).

### Affected coronary arteries

After excluding the two patients who did not undergo coronary angiography, descriptive analysis was done to the remaining 16 patients. Most of those (10, 62.5%) had single vessel disease, which is the typical presentation. However, association of Wellens syndrome with two (4, 25%), and three (2, 12.5%) vessels involvement may constitute an atypical encounter (Table 2). The presence of multiple vessel disease outcome at coronary angiography was recently studied, and reported, providing evidence that Wellens syndrome may present with multiple coronary artery involvement [33, 34].

The most frequently affected coronary artery part was the mid LAD artery, representing 50% of those patients. Proximal LAD artery involvement was present in only 25% of the patients. This deviated from the classical finding in previous studies that suggests proximal LAD involvement as the most common [1, 2, 4, 18, 21], in spite of being the same artery. Relevant to mention that mid LAD artery involvement was encountered in a previous study as well [4]. Furthermore involvement of none isolated right coronary artery and the left circumflex artery represented 25 and 18.8% respectively. Both arteries’ involvement was previously reported [32]. Right coronary artey involvement, particularily the proximal part, was previously found in an atypical presentation, where the classical type A biphasic t wave appeared in inferior ECG leads [35].

It is important to mention that four (25%) out of Wellens syndrome patient who underwent coronary angiography had normal coronary arteries (Table 2). This supports previous finding in that not all patients who fulfill Wellens syndrome criteria have coronary artery disease [18]. And Wellens syndrome though highly specific, its specificity does not reach 100% [21]. Moreover, atypical angiographic variation other than atherosclerosis, such as myocardial bridging, was previously reported in association with typical type A wellens syndrome [36].

Six type A Wellens syndrome patients (out of 16, 37.5%) had mid LAD involvement. Although there is no statistically significant association between Wellens syndrome type and acute coronary syndrome (Table 3), this finding may suggest that mid LAD involvement may be encountered in type A Wellens more than type B, though not statistically significant. Moreover, the study of Wellens syndrome type in association with coronary angiographic findings was not frequently encountered in the literature. A recent article [37], highlighted by the American College of Cardiology, suggested a nearly similar association of both types with LAD atery involvement.

## Limitations

This descriptive cross sectional study was conducted in one cardiac centre that despite being a highly selective center of referral for many state hospitals, limits the number of encountered patients who fulfilled the inclusion criteria. Furthermore, the statistical methods used on the small batches, namely Wellens syndrome patients, different types and risk factors subgroup analysis, may produce doubtful statistical significance. Hence, extending the period as well as expanding study area, and sample size, with supported facilitation would relieve some limitations that might have anckered producing more statistically significant results, considering the limited numbers of patients who fulfilled Wellens syndrome criteria. Furthermore, considering each coronary artery involvement as an isolated variable within multiple vessel disease patients, who had Wellens syndrome, during statistical analysis sheds light on specific culprit association. However, this may weaken overall significance.

## Conclusion

Despite being relatively uncommon, Wellens syndrome is a serious condition that is not classified as a rare condition, as it complicates 15% of acute coronary syndrome patients with a 55.6% possibility of becoming type A. It can be encountered in any acute coronary syndrome patient with the exclusion of STEMI, either NSTEMI or unstable angina, being more prevalent in NSTEMI patients. Type A Wellens syndrome may be encountered more frequently than type B.

Wellens syndrome may complicate acute coronary syndrome even without identifiable risk factors. Diabetes was the most frequently encountered risk factor in Wellens syndrome patients. However, no specific risk factor had statistically significant association.

Coronary angiographic findings other than the involvement of the proximal part of the left anterior descending artery may present. The most frequently encountered in this study was the mid part of the left anterior descending artery. However, it is possible for patients who fulfill Wellens syndrome criteria to have a normal coronary angiography regardless of Wellens syndrome type and presence of risk factors.

## Perspectives

Awareness should be raised towards early identification of Wellens syndrome by practicing physicians and health care providers in order to reduce the number of missed cases and thus preventing the development of irreversible consequences.

It is acceptable to mention that, given the proven prevalence in this as well as previous studies, further studies of Wellens syndrome should focus on the condition as inclusion criteria of research. This would allow more in depth analysis of its pattern of incidence. A larger sample size might need a more prolonged period of time to attain an acceptable representation of any given population, but would ultimately produce statistically significant results that can help in answering questions this study could not.

Results of this study suggest that Wellens syndrome does not have a specific risk factor pattern for prediction despite finding risks more frequent than others. However, given its limitations it is safe to recommend that a more focused approach to Wellens syndrome risk factors should be implemented taking into account population variation of acute coronary syndrome risk factors.

Reasons behind coronary angiographic variation in Wellens syndrome could not be explained well despite being in line with previous findings of specificity. Therefore, inclusion of Wellens syndrome type variation beside coronary angiographic findings on a larger scale may help define pattern of presentation.

Development of scoring systems for either prediction or prognostication of Wellens syndrome may potentially play a role in early recognition and management. Therefore, further directed studies are warranted. Furthermore, use of artificial intelligence was recently explored as a potential.

Furthermore, the emerging use of artificial intelligence (AI), and machine learning within the field of cardiology, particularly in conditions related to coronary artery disease, sheds light on its potential role in rapid prediction and management, given the detrimental implications of missed diagnosis that may occur in such conditions. While AI-enabled ECG records were previously proven beneficial for diagnosis and management of chronic coronary artery disease, newer evidence suggests an identification and prognostication accuracy of STEMI competing with that of established scoring systems [38]. Thus, supporting the need for further studies on utilisation of artificial intelligence in recognition and management of Wellens syndrome.

### Supplementary Information

**Additional file 1.** Questionnaire.

## Data Availability

The datasets generated during and/or analysed during the current study are available from the corresponding author on reasonable request.

## Abbreviations

ACS: Acute coronary syndrome
DM: Diabetes mellitus
ECG: Electrocardiogram
LAD: Left anterior descending
LCx: Left circumflex
MACE: Major adverse cardiac event
NSTEMI: None ST elevation myocardial infarction
RCA: Right coronary artery
SPSS: Statistical package for the social sciences
STEMI: ST elevation myocardial infarction
