# Supplementary material for: Wellens Syndrome: prevalence, risk factors and coronary angiographic variation. A cross-sectional study
Source: BMC Cardiovasc Disord. 2024 Feb 1;24:84. doi: 10.1186/s12872-024-03752-y (PMC10832078; doi:10.1186/s12872-024-03752-y)
Supplement: Supplementary file 1 — Additional file 1. Questionnaire. [file 12872_2024_3752_MOESM1_ESM.docx]

Wellens Syndrome: Prevalence, Risk Factors and Coronary Angiographic Variation. A Cross-Sectional Study

Questionnaire

This is a questionnaire for a study about risk factors and coronary angiographic findings in Ahmed Gasim hospital patients with Wellens syndrome, conducted by Sami Mohamed et al. Please answer all the numbered questions and make sure to use the consent form provided. You can use the attached paper about Wellens criteria.

1. Age (choose one only):

20-30 years ( ) 30-40 years ( ) 40-50 years ( )

50-60 years ( ) 60-70 years ( ) 70-80 years ( )

more than 80 ( )

1. Gender: Male ( ) Female( )
2. BMI (choose one only):

Less than 25 ( ) 25-30 ( ) more than 30 ( )

1. Choose yes for any of the following risk factors if confirmed present:

Hypertension: Yes ( ) No ( )

Diabetes: Yes ( ) No ( )

Hyperlipidemia Yes ( ) No ( )

Family history of Coronary Artery Disease: Yes ( ) No ( )

Previous coronary stent/balloon revascularization: Yes ( ) No ( )

Chronic kidney disease: Yes ( ) No ( )

Smoking now or previously Yes ( ) No ( )

1. ECG finding prior to angiography and recorded during a pain free period (choose one only):

Wellens type A - inverted T wave in V2-V3 ( )

Wellens type B - biphasic T wave in V2-V3 ( )

1. Other Ischemic ECG changes apart from Wellens: Yes ( ) No ( )
2. Coronary angiography findings - artery affected

(you can choose more than one):

Normal coronary arteries ( )

Left main coronary artery ( )

Proximal left anterior descending artery ( )

Mid left anterior descending artery ( )

Distal left anterior descending artery ( )

Diagonal artery ( )

Left circumflex artery ( )

Right coronary artery ( )

1. Coronary angiography findings – conclusion (choose one only):

Normal coronary angiography ( )

Single vessel disease ( )

Dual vessel disease ( )

Three vessel disease ( )

Attached to Questionnaire

The following are the required criteria for the diagnose of Wellens syndrome:

Prior history of chest pain.

During chest pain: ECG is normal or with mild ST elevation or depression, or with terminal negative deflection of the T wave in V_1_ and V_2_.

Cardiac enzymes are normal or mildly elevated.

No pathologic precordial Q-waves.

No loss of precordial R-waves.

Deeply inverted or biphasic T-waves in V2 when pain free.

The following is an ECG example for wellens syndrome:


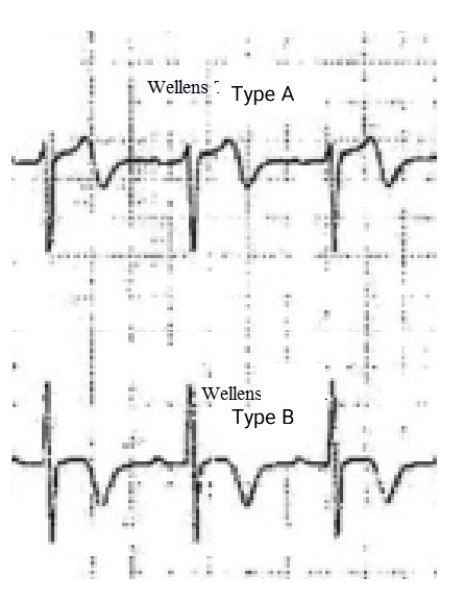


Mead NE, O’Keefe KP. Wellen’s syndrome: An ominous EKG pattern. J Emerg Trauma Shock [Internet]. 2009 Sep [cited 2017 Feb 25];2(3):206–8. Available from: http://www.ncbi.nlm.nih.gov/pubmed/20009314
